# Propolis as a Cariostatic Agent in Lozenges and Impact of Storage Conditions on the Stability of Propolis

**DOI:** 10.3390/pharmaceutics15061768

**Published:** 2023-06-19

**Authors:** Anna Kurek-Górecka, Paweł Ramos, Małgorzata Kłósek, Elżbieta Bobela, Zenon P. Czuba, Radosław Balwierz, Paweł Olczyk

**Affiliations:** 1 Department of Community Pharmacy, Faculty of Pharmaceutical Sciences in Sosnowiec, Medical University of Silesia in Katowice, Kasztanowa 3, 41-200 Sosnowiec, Poland; polczyk@sum.edu.pl; 2 Department of Biophysics, Faculty of Pharmaceutical Sciences in Sosnowiec, Medical University of Silesia in Katowice, Jedności 8, 41-200 Sosnowiec, Poland; pawelramos@sum.edu.pl; 3 Department of Microbiology and Immunology, Faculty of Medical Sciences in Zabrze, Medical University of Silesia in Katowice, Jordana 19, 41-808 Zabrze, Poland; mklosek@sum.edu.pl (M.K.); ebobela@sum.edu.pl (E.B.); zczuba@sum.edu.pl (Z.P.C.); 4 Institute of Chemistry, University of Opole, Oleska 48, 45-052 Opole, Poland; radoslaw.balwierz@uni.opole.pl

**Keywords:** propolis, antibacterial effect, oral cavity, lozenges, storage conditions

## Abstract

Propolis is known as a source of compounds with strong antibacterial activity. Due to the antibacterial effect against streptococci of the oral cavity, it seems to be a useful agent in decreasing the accumulation of dental plaque. It is rich in polyphenols which are responsible for a beneficial impact on the oral microbiota and antibacterial effect. The aim of the study was to evaluate the antibacterial effect of Polish propolis against cariogenic bacteria. The minimum inhibitory concentration (MIC) and minimum bactericidal concentration (MBC) were determined on cariogenic streptococci related to the occurrence of dental caries. Lozenges based on xylitol, glycerin, gelatin, water, and ethanol extract of propolis (EEP) were prepared. The effect of prepared lozenges on cariogenic bacteria was assessed. Propolis was compared to chlorhexidine which is used in dentistry as the gold standard. In addition, the prepared propolis formulation was stored under stress conditions to assess the influence of physical conditions (i.e., temperature, relative humidity, and UV radiation). In the experiment, thermal analyses were also performed to evaluate the compatibility of propolis with the substrate used to create the base of lozenges. The observed antibacterial effect of propolis and prepared lozenges with EEP may suggest directing subsequent research on prophylactic and therapeutic properties decreasing the accumulation of dental plaque. Therefore, it is worth highlighting that propolis may play an important role in the management of dental health and bring advantages in preventing periodontal diseases and caries as well as dental plaque. The colorimetric analyses carried out in the CIE L*a*b* system, microscopic examinations, and TGA/DTG/c-DTA measurements indicate the unfavorable effect of the tested storage conditions on the lozenges with propolis. This fact is particularly evident for lozenges stored under stress conditions, i.e., 40 °C/75% RH/14 days, and lozenges exposed to UVA radiation for 60 min. In addition, the obtained thermograms of the tested samples indicate the thermal compatibility of the ingredients used to create the formulation of lozenges.

## 1. Introduction

Propolis is a natural substance with strong antibacterial activity; therefore, it may be potentially useful in dentistry and oral health management. The ethanolic extract of propolis exhibits antibacterial activity against *Streptococcus mutans* and *Lactobacillus* spp. Which are responsible for biofilm formation. In addition, among the cariogenic bacteria, there are bacteria associated with the initiation and development of dental caries as follows: *Enterococcus faecalis*, *Actinomyces* spp., *Rothia dentocariosa, Bifidobacterium* spp., *Scardovia* spp., *Prevotella* spp., and *Veillonella* spp. [1].

The mechanisms of antimicrobial action of ethanolic extract of propolis are connected with changes in the cytoplasmatic membrane and cell wall, resulting in pore formation in cell membranes. This, in turn, leads to membrane rupture, partial bacteriolysis, and the formation of pseudo-multicellular colonies. Moreover, there have been observations of an interaction between the hydrophobic part of the membrane and the polar group of propolis. The ethanolic extract of propolis also exhibits the ability to inhibit protein synthesis [2].

Researchers underline that phenolic compounds are responsible for the antibacterial action of propolis. Higher concentrations of phenolic acids and flavonoids exhibit stronger antibacterial activity. Among the polyphenols, t-farnesol, apigenin, galangin, pinocembrin, pinostrobin, artepillin C, baccharin, quercetin, and caffeic acid phenethyl ester (CAPE) demonstrate antibacterial effect [3,4,5].

Therefore, propolis may be beneficial in the development of dosage forms intended for oral cavity use. Pre-formulation research holds significant importance in the field of pharmacy. It enables the early detection of irregularities in the designed drug formulation, ultimately saving both time and money.

Pre-formulation tests encompass compatibility tests of the ingredients used in the formulation, as well as aging tests conducted under stressful conditions. These tests involve subjecting the formulation to short-term exposure to extreme conditions, such as a temperature range of 40 °C to 70 °C and a relative humidity (RH) of 75%, typically for a duration of 14 days [6,7]. Additionally, pre-formulation tests include the assessment of the formulation’s response to UV radiation [7,8].

This assessment is crucial because the influence of UV radiation during the photolysis process can lead to the decomposition of both the active pharmaceutical ingredient (API) and the excipients used in the formulation. This decomposition can significantly impact the stability of the formulation.

Thermal analyses play a crucial role in assessing the impact of physical conditions, such as temperature, relative humidity, or UV radiation on the compatibility between the API and excipients [7,9,10] Techniques such as thermogravimetry (TGA) and differential thermal analysis (DTA) are commonly employed in these analyses due to their numerous advantages. These techniques offer benefits such as ease of sample preparation, low measurement costs, and minimal sample requirements, and fast, reliable, and repeatable results. Consequently, they are widely utilized in the pharmaceutical industry [11,12].

Considering the beneficial impact of propolis on the oral cavity, particularly its antibacterial effect, we conducted a study to prepare lozenges with ethanolic extract of propolis. Our objective was to evaluate the antibacterial action of Polish propolis against cariogenic bacteria, as well as the stability and susceptibility of the prepared lozenges with propolis under different storage conditions. Additionally, we aimed to assess the potential thermal and photolytic degradation of the lozenges and the ethanolic extract of propolis. It is important to note that physical conditions during storage may lead to the deactivation of the biologically active substances responsible for the antibacterial effect of propolis. Therefore, we conducted antimicrobial analysis on the EPP, the lozenges with EEP, and the lozenges with EEP after storage under stress conditions.

## 2. Materials and Methods

### 2.1. Materials

The Polish propolis sample was obtained from Kamianna, located in southern Poland. Ethanol 96% was purchased from POCH (Gliwice, Poland). Disodium phosphate, Tween 20, xanthan gum, citric acid monohydrate, xylitol, glycerin, gelatin were purchased from Sigma-Aldrich (Steinheim, Germany). DMSO was purchased from Sigma (St. Louis, MO, USA). 2% solution of chlorhexidine digluconate was obtained from Chemidental (Pabianice, Poland). The bacterial strains used in the study were obtained from the American Type Culture Collection (ATCC, Manassas, VA, USA). The strains included *Streptococcus mutans* ATCC 33535, *Streptococcus salivarius* ATCC 13419, *Streptococcus mitis* NC IMB 13770, and *Streptococcus oralis* ATCC 6249.

### 2.2. Preparation of Ethanol Extract of Propolis

A total of 20 g of Polish propolis (POL) was mechanically ground and then added to 100 mL of 70% ethanol (*w*/*v*) for extraction. The mixture was stored in a dark environment at room temperature for 7 days. After extraction, the mixture was filtered under vacuum, and the residue was subjected to re-extraction using another 100 mL of 70% ethanol (*w*/*v*) under the same conditions. The extract and re-extract were combined and stored at 4 °C for 24 h to allow the wax to precipitate. Subsequently, the mixture was filtered and the filtrate was evaporated and dried under vacuum at 40 °C until a solid form was obtained. The extraction efficiency, expressed as a percentage, was calculated by dividing the mass of the solid extract obtained by the initial mass of the raw propolis used. The extraction efficiency was determined to be 54%.

A final concentration of the extract solution at 500 mg/mL was prepared by dissolving 5.0 g of the solid extract in 10.0 mL of 70% ethanol (*w*/*v*). The composition of the solid ethanol extract of Polish propolis has been previously described by Kurek-Górecka et al. [13].

#### Preparation of Ethanol Extract of Propolis for Microbiological Assay

The dried extract of propolis was dissolved in DMSO to prepare various final concentrations: 0.78 µg/mL, 1.56 µg/mL, 3.125 µg/mL, 6.25 µg/mL, 12.5 µg/mL, 25 µg/mL, 50 µg/mL, and 100 µg/mL.

### 2.3. Preparation of Lozenges with EEP

The lozenges were prepared using a gelatin–glycerol mixture. To prepare the lozenges, 20 g of gelatin, 48 g of glycerin, 20 g of xylitol, and 10 g of water were used. The glycerol and xylitol were mixed to form a syrup, to which gelatin was added. The mixture was left for 15 min to swell, then heated at 90 °C to dissolve the gelatin. Once a uniform mass was obtained, 2 g of prepared 50% ethanol extract of propolis was added. The resulting mass was poured into lozenge molds, and lozenges weighing 2.5 g were formed.

Each prepared lozenge was dissolved in 50 mL artificial saliva. The artificial saliva used in this study was prepared according to Gittings’s method, without mucin and amylase [14]. The composition of the artificial saliva was as follows: 9.009 mL buffer pH 7.4, 5.6 µL Tween 20, 0.08 (*w*/*v*%) xanthan gum. The buffer was prepared by combining 91.3 mL disodium phosphate (28.36 g/L) and 8.7 mL citric acid monohydrate (21.00 g/L). Deionized water was added to all components to make up a total volume of 100 mL. Finally, the pH was adjusted to pH 7.4 using 1 M HCl [15].

### 2.4. Influence of Physical Factors on Tested Samples

#### 2.4.1. Higher Temperature and Relative Humidity

The propolis lozenges were subjected to stress conditions by storing them in an incubator with controlled temperature and relative humidity. The lozenges were placed on Petri dishes and stored at a temperature of 40 °C (±2 °C) and a relative humidity (RH) of 75% (±5%) for a duration of 14 days [6,16]. The incubator used for this purpose was a Memmert company air circulation incubator (Schwabach, Germany) which maintained constant parameters.

#### 2.4.2. Ultraviolet Irradiation

The propolis lozenges were subjected to UVA (315–400 nm) radiation [8,16]. The irradiation was conducted using a Medison 250 lamp manufactured by Schulze & Bohm (Brühl, Germany). The lamp consisted of four radiators, each with a power of 20 W. The tested samples were exposed to ultraviolet irradiation from a distance of 30 cm. Two different durations of UVA exposure were used in the experiment: 30 and 60 min.

### 2.5. Profile of Thermal Decomposition

The thermal stability of propolis lozenges and lozenge base was determined using thermogravimetric analysis (TGA). The study employed Thermogravimeter TG 209 F3 Tarsus by Netzsch (Selb, Germany). Thermogravimetric dynamic analysis was performed on the tested samples, and various curves were recorded, including thermogravimetry (TG), first derivative (DTG), second derivative D2TG, and calculated differential thermal analysis (c-DTA) curves. The recorded curves were obtained for 10 mg of propolis lozenges at a heating rate of 10 K/min. in the temperature range of 35–600 °C under a nitrogen atmosphere (total flow nitrogen—40 mL/min.). The measurements were conducted using Al_2_O_3_ crucibles [17,18].

Differential thermal analysis (c-DTA) was performed to calculate the endothermal and exothermal events for all samples. In this method, a multiple-point temperature calibration was carried out using c-DTA. To achieve this, the onset temperatures of the melting peaks of high-purity reference materials (In, Sn, Zn, Al, BaCO_3_, and Au) were determined across the entire temperature range [17].

The thermogravimetric curves obtained were analyzed using Proteus 8.0 software by Firm Netzsch (Selb, Germany).

### 2.6. Colorimetric Analysis in CIE L*a*b* System

Colorimetric analysis in the CIE L*a*b* system was performed for the tested propolis lozenges. The colorimeter NH 310, produced by 3nh company (China), was used in the study. Analyses of colour parameters were conducted for propolis lozenges that were both not subjected to and exposed to stress conditions such as higher temperature/relative humidity and UVA irradiation. All measurements were performed eight times for each sample, and the resulting values were averaged. 

The parameters: L* (lightness), a* (redness), and b* (yellowness) were used to ana-lyse changes in the CIE L*a*b* colour space of the propolis lozenges. Parameter L* takes values from 0 to 100. Value 0 indicates the black colour, and value 100 indicates the white colour. Shades of grey take values from 1 to 99. Parameter a* indicates the redness of the samples. The positive and negative value of a* indicates the domination of red or green colour, respectively. Parameter b* indicates the yellowness of the samples. Negative and positive b* suggests the domination of blue colour or yellow colour, respectively [7,19,20].

For storage in different physical conditions, propolis lozenges were calculated for total colour difference (ΔE*) parameter according to Formula (1) [7,19].

ΔE* = [(ΔL*)^2^ + [(Δa*)^2^ + [(Δb*)^2^]^1/2^, (1)

where:

ΔL*—represents the mean difference in brightness between the initial propolis lozenges and those exposed to stress conditions.

Δa*—represents the mean difference in redness between the initial propolis lozenges and those exposed to stress conditions. 

Δb*—represents the mean difference in yellowness between initial propolis lozenges and those exposed to stress conditions. 

The browning index (BI) was calculated for the tested samples according to Formula (2) using the parameters obtained for CIE L*a*b* measurements [7]:

BI = 100[(x − 0.31)]/0.172,(2)

where parameter x was calculated according to Formula (3):

x = (a* + 1.75 L*)/(5.645 L* + a* − 3.012 b*),(3)

### 2.7. Optical Microscopy Images

An optical microscope equipped with a camera 5MPX Opta-Tech company (Poland) was used in the study. The magnification of the recorded images (objective × eyepiece) was 4×, 10×, and 40× with numerical apertures 0.25 and 0.65, respectively. The photos for the initial propolis lozenge, lozenge base, and the propolis lozenges exposed to tested physical stored conditions were registered. Quantitative analysis was carried out for the images obtained at 40× magnification. For this purpose, three preparations were made from each sample. The observation was carried out in five fields for each image.

### 2.8. Bacterial Strains

In the experiment, the bacterial strains used were obtained from the American Type Culture Collection (ATCC, Manassas, VA, USA). The specific strains employed were as follows: *Streptococcus mutans* ATCC 33535, *Streptococcus salivarius* ATCC 13419, *Streptococcus mitis* NC IMB 13770, *Streptococcus oralis* ATCC 6249. Bacteria were grown on blood agar with 5% CO_2_ at 37 °C.

### 2.9. Determination of the Minimum Inhibitory Concentration and Minimum Bactericidal Concentration of EEP

In this study, the antimicrobial activity of the ethanolic extract of Polish propolis was evaluated using the minimum inhibitory concentration (MIC) assay. The MIC is the lowest concentration of antimicrobial agent that completely inhibits the growth of organism in microdilution wells or in tubes after 24 h of incubation at 37 °C. In our experiments, MIC was evaluated using the microdilution method in 96-well microtiter plates. The dry ethanol propolis extract was dissolved in dimethyl sulfoxide (DMSO, Sigma, St. Louis, MO, USA). Serial dilutions of the extract were then performed to obtain different final concentrations: 100, 50, 25, 12.5, 6.25, 3.125, 1.56, 0.78 µg/mL). For each bacterial strain (*Streptococcus mutans*, *Streptococcus salivarius*, *Streptococcus mitis*, and *Streptococcus oralis*), a bacterial inoculum of 0.5 McFarland turbidity was prepared by suspending colonies in 0.85% NaCl solution. Brain Heart Infusion (BHI) broth was used as the growth medium for all bacterial species. The examined wells contained 160 µL of appropriate broth, 20 µL of the ethanolic extract of propolis, and 20 µL of a bacterial suspension at the final concentration of 1 × 10^5^ CFU/mL. Sterility control and growth control as well as 0.2% solution of chlorhexidine digluconate (Chemidental, Pabianice, Poland) were also included for comparison. Chlorhexidine (CHX) is considered a gold standard and is commonly used as an antimicrobial and antiplaque agent due to its bactericidal activity. A 0.2% solution of chlorhexidine digluconate (Chemidental, Pabianice, Poland) was prepared. The positive control consisted of a 0.2% solution of CHX incubated with appropriate species of streptococci. BHI without appropriate species of streptococci served as clarity control. The negative control involved the appropriate species of streptococci in Brain Heart Infusion broth. The MIC value of the propolis extract was determined after 24 h of incubation at 37 °C by analyzing spectrophotometric absorbance at 405 nm wavelength in Eon Microplate Spectrophotometer (BioTek, Winooski, VT, USA). 

For the minimum bactericidal concentration (MBC assessment), 20 µL of liquid culture that showed no visible growth in the MIC assay was taken and inoculated onto fresh blood agar plates. The plates were then incubated at 37 °C with 5%CO_2_ for 24 h. The minimum bactericidal concentration was determined as the least concentration showing no visible growth on plates [21,22].

### 2.10. Determination of Minimum Inhibitory Concentration and Minimum Bactericidal Concentration of Lozenges with Propolis

To determine the antibacterial properties of propolis lozenges, each lozenge, including those with propolis and those subjected to different aging factors (higher temperature and relative humidity, UVA for 30 min, and UVA for 60 min), was dissolved in 50 mL of artificial saliva. We used lozenges with propolis and lozenges that had been subjected to (1) higher temperature and relative humidity, (2) UVA for 30 min, and (3) UVA for 60 min (described in Section 2.4) in order to determine aging factors. 

To ensure sterility, the lozenges and artificial saliva were autoclaved at 121 °C for 15 min. Each well of 96-well plate contained 200 μL of saliva with dissolved lozenges and with appropriate bacterial species. The final concentration of propolis was 100 µg/mL and the final concentration of bacterial suspension was 1 × 10^5^ CFU/mL. Artificial saliva with appropriate bacterial species, but without the lozenge, served as positive controls whereas artificial saliva alone as negative controls. The MIC value of lozenges was determined after 24 h of incubation at 37 °C by analyzing spectrophotometric absorbance at 405 nm wavelength in Eon Microplate Spectrophotometer (BioTek, Winooski, VT, USA). 

For the minimum bactericidal concentration—MBC assessment, 20 µL of liquid culture was taken from each well that showed no visible growth and further cultured on fresh blood agar plates followed by incubation at 37 °C with 5%CO_2_ for 24 h. The minimum bactericidal concentration was determined as the least concentration showing no visible growth on plates [22].

### 2.11. Statistical Analysis

The mean (±SD) of eight colorimetric measurements of a single sample were analyzed with one-way ANOVA to access statistical significance (*p* < 0.05). For the calculations, the Statistica software by TIBCO Software Inc. (Palo Alto, CA, USA) was used.

The minimal inhibitory concentration (MIC) and the minimum bactericidal concentration (MBC) were performed in triplicate.

## 3. Results and Discussion

In this study, the ethanolic extract of propolis originating from Poland was obtained with two-step extractions. Polish propolis belongs to the poplar type. The flavonoid profile of the Polish extract of propolis is similar to *Populus nigra* buds. The polyphenols responsible for the antibacterial action include: galangin, chrysin, pinobanksin, apigenin, caffeic acid, cinnamic acid, and gallic acid [13,23]. In order to determine the antibacterial activity of the ethanolic propolis extract against cariogenic bacteria, four species of bacteria, which included *Streptococcus mutans*, *Streptococcus salivarius*, *Streptococcus mitis*, and *Streptococcus oralis*, were used. The prepared ethanolic extract of propolis at the range of 25–100 µg/mL demonstrated in vitro antibacterial activity. After evaluating the minimum inhibitory concentration (MIC) and minimum bactericidal concentration (MBC) for the four reference strains, the lozenges with EEP were prepared. In recent years, there has been a growing trend in the use of natural products such as propolis for the treatment and prevention of oral bacterial diseases, leading to numerous conducted clinical trials and experimental studies [24,25,26,27,28,29]. Therefore, the prepared lozenges might be helpful in the maintenance of the oral cavity and in biofilm reduction. Dziedzic et al. showed the antibacterial effect of Polish propolis ethanol extract [30]. Therefore, the prepared propolis formulation was stored under stress conditions to assess the influence of temperature, relative humidity, and UV radiation to evaluate the compatibility of propolis with the substrate used to create the base of lozenges and compare the antibacterial effect of propolis and prepared lozenges with EEP. The influence of different storage conditions on antibacterial effect of propolis is important to create the new formulation of propolis for oral cavity. 

### 3.1. Prepared Propolis Lozenges

**Figure 1:**
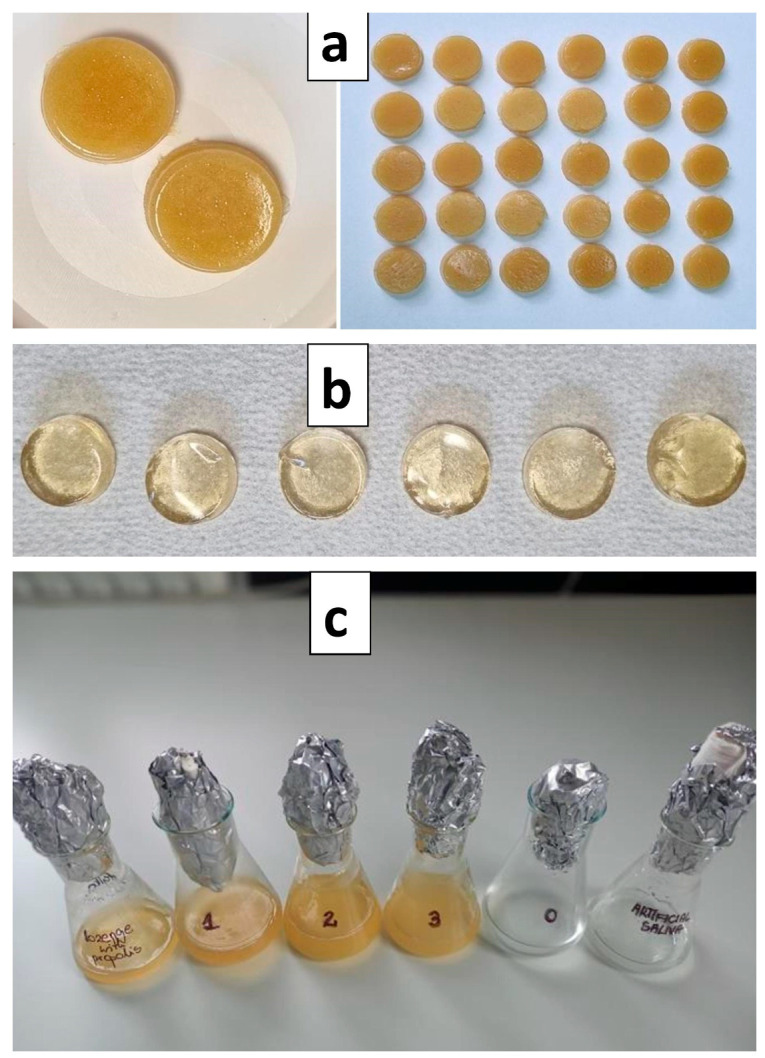
Lozenges with propolis extract (**a**), without propolis extract (**b**), and lozenges dissolved in artificial saliva (**c**).

The prepared lozenges weighed 2.5 g ± 0.09. Each lozenge contained 25 mg of EEP. Additionally, the lozenges without EEP were prepared. The visual aspect of prepared lozenges is presented in Figure 1a,b.

Each lozenge was dissolved in artificial saliva to obtain the concentration of 100 µg/mL (Figure 1c).

Chewable lozenges were proposed as dosage form because during sucking, propolis lozenges can be dispersed throughout the mouth, where it exerts its antibacterial effect. The antibacterial substances contained in propolis, such as apigenin, galangin, pinocembrin, pinostrobin, artepillin C, baccharin, quercetin, caffeic acid phenethyl ester should be released slowly in the mouth. The developed formulation should be stable—without losing its properties during storage, which is why tests aimed at assessing susceptibility to decomposition caused by heat and light were carried out.

All prepared lozenges were tested under stressful conditions because components of propolis may be unstable and susceptible to high temperature, humidity, air oxygen, and UV radiation. The factors mentioned above are well-known agents that cause the degradation of compounds containing double bonds such as polyphenols [31]. Arruda et al. demonstrated the formation of three isomers of artepillin C and one from *p*-coumaric acid during physical factors such as light and high temperature (50 °C). The stability studies showed that the presence of light and temperature of 40 °C, as well as oxygen, did not change the content of *p*-coumaric acid. However, the same conditions significantly influenced the content of artepillin C [32]. Therefore, during stress conditions, we should take into consideration the possibility of degradation of active compounds of propolis. Consequently, it may lead to changes in antibacterial properties. Therefore, the impact of stress conditions on the prepared lozenges and microbiological analysis was examined. 

#### 3.1.1. Thermal Decomposition Behavior and Thermal Compatibility of Ingredients of the Formulation

**Figure 2:**
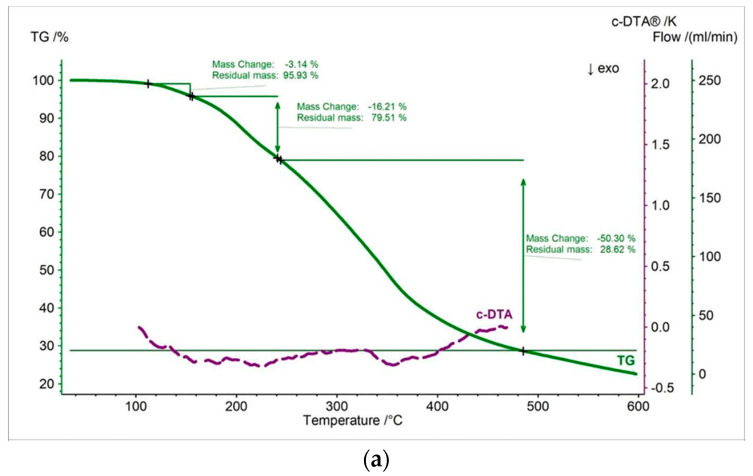

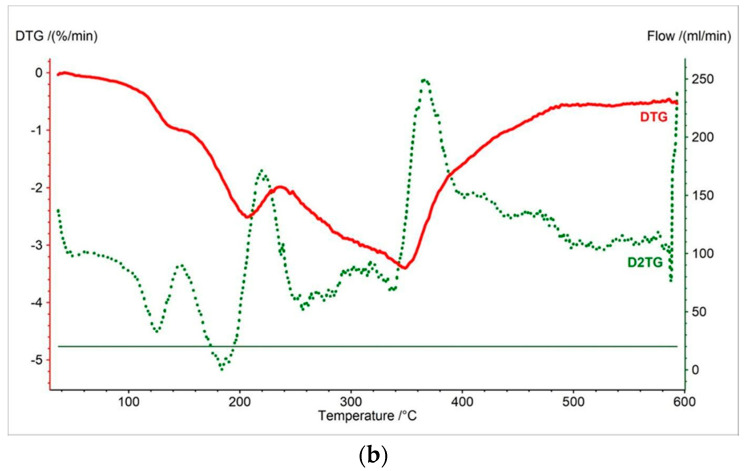
(**a**) TG, c-DTA and (**b**) DTG, D2TG curves of pure propolis.

**Figure 3:**
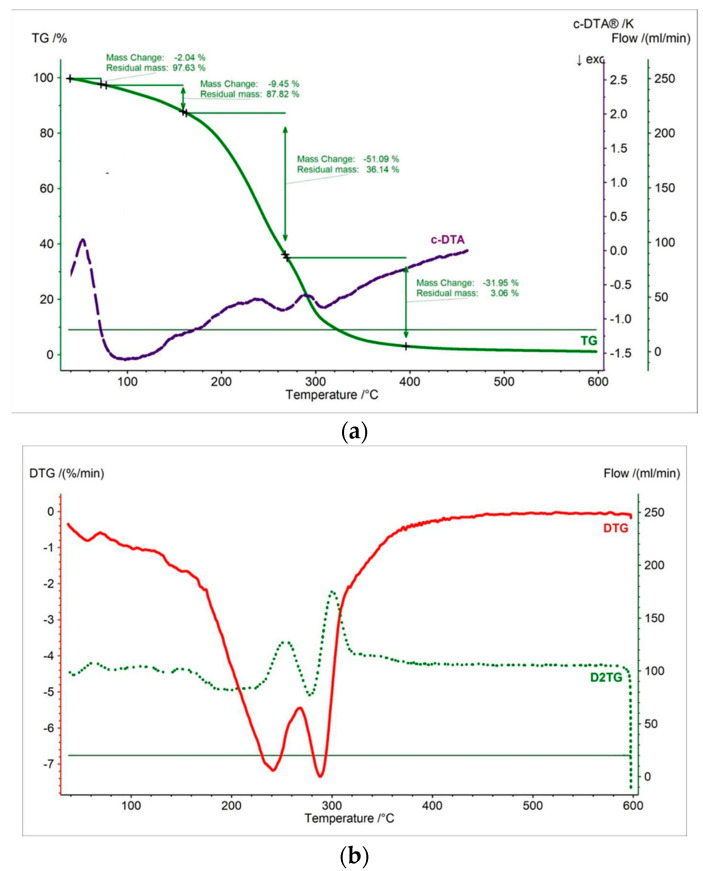
(**a**) TG, c-DTA and (**b**) DTG, D2TG curves of lozenge base.

**Figure 4:**
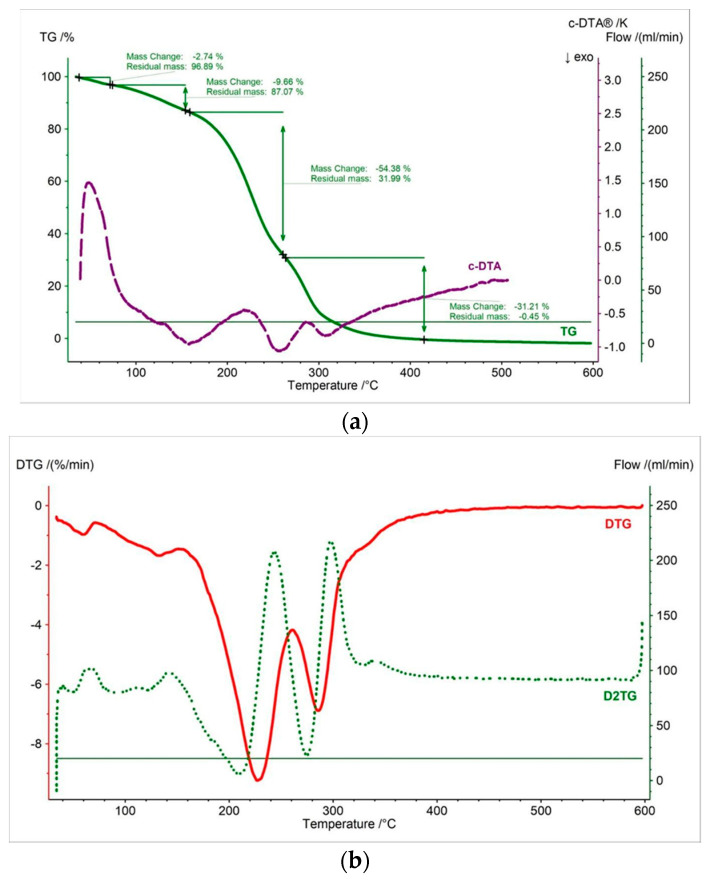
(**a**) TG, c-DTA and (**b**) DTG, D2TG curves of propolis lozenges.

**Figure 5:**
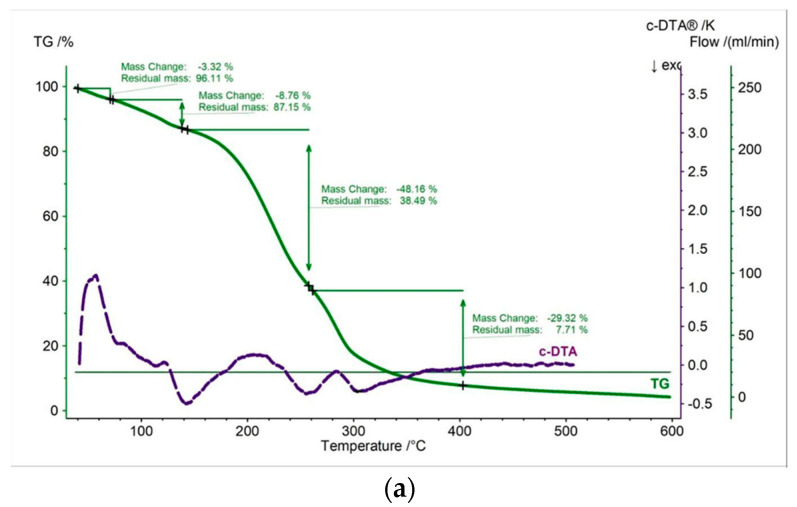

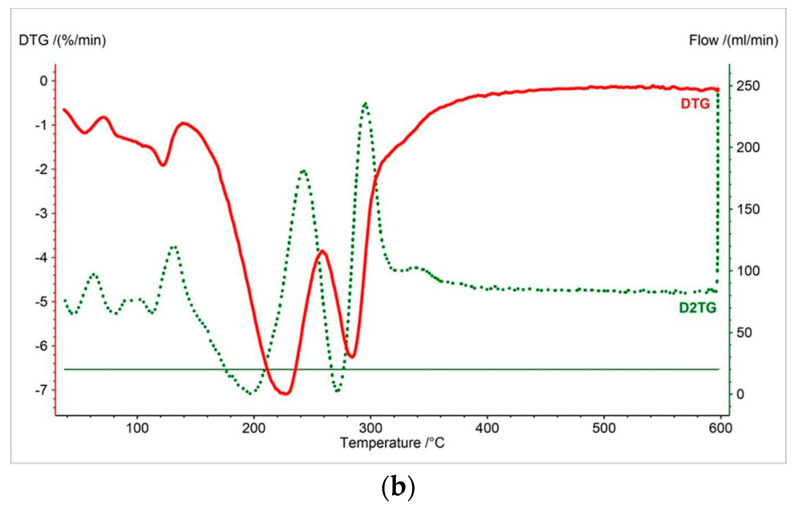
(**a**) TG, c-DTA and (**b**) DTG, D2TG curves of propolis lozenges exposed to 40 °C/75%HR/14 days.

**Figure 6:**
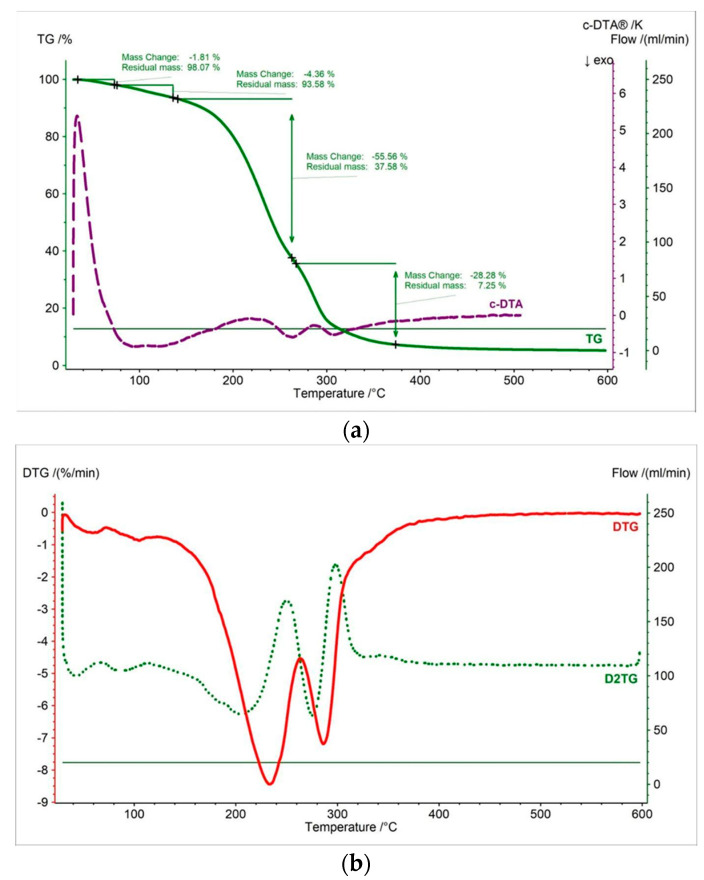
(**a**) TG, c-DTA and (**b**) DTG, D2TG curves of propolis lozenges exposed to UV/30 min.

**Figure 7:**
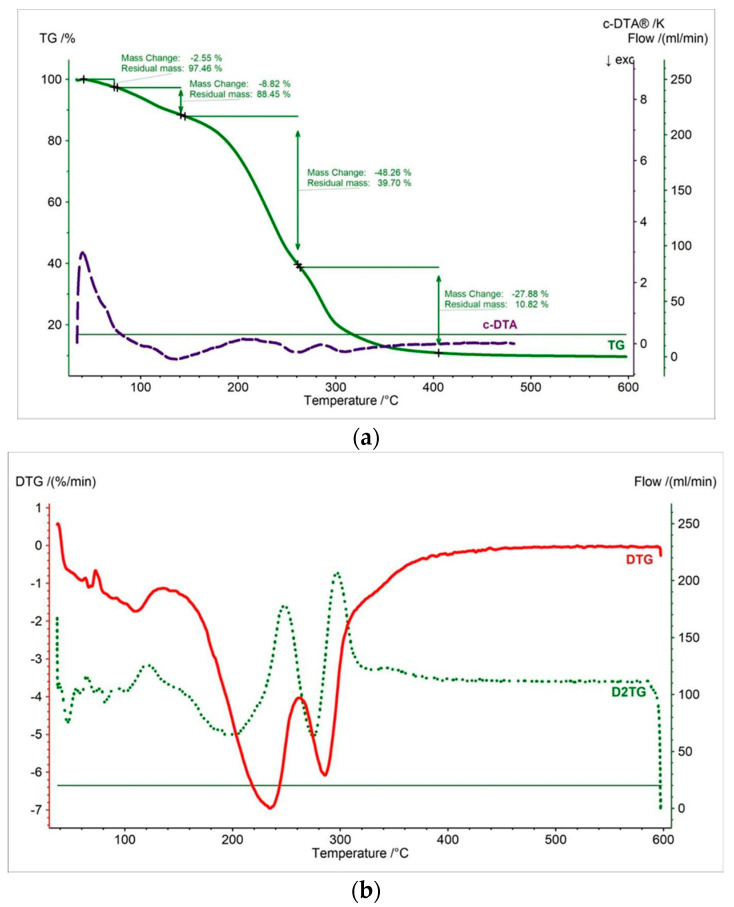
(**a**) TG, c-DTA and (**b**) DTG, D2TG curves of propolis lozenges exposed to UV/60 min.

In this work, thermal measurements were performed for all tested samples. In Figure 2, Figure 3, Figure 4, Figure 5, Figure 6 and Figure 7 and Table 1, Table 2 and Table 3 are presented the thermogravimetric profiles and events of the pure propolis, base of lozenges, and initial propolis lozenges treated with physical factors. In addition to the TG, DTG, and D2TG curves, c-DTA curves were recorded to visualize exo- and endothermic phenomena.

Pure propolis has three decomposition steps (Figure 2, Table 1). The first stage presented in the DTG curve begins at 67 °C and ends at 153 °C with a maximum mass change at 145 °C (Figure 2b, Table 2). The first stage is accompanied by a relatively small weight loss of 3.14% (Figure 2a). This stage is probably connected with a loss of volatile compounds [33]. The second stage starts at 153 °C and has a maximum mass loss at 206 °C. The last stage occurs in the temperature range of 236 °C to 491 °C, with a maximum mass change, shifted towards higher temperature at 348 °C (Figure 2b, Table 2). Additionally, exothermic effects were recorded on the c-DTA for the second and third stages, showing a maximum peak at 226.5 °C and 355.6 °C. This confirms that the mixture of organic components present in the tested propolis undergoes decomposition during these stages [33,34,35].

TGA, DTG, and D2TG curves of the lozenge base as shown in Figure 5 indicate that the decomposition process began at 189.1 °C and consisted of four stages (Table 1). The first mass loss of the DTG occurred in the temperature range of 33 °C to 70 °C, with a maximum peak at 56 °C (Figure 3b, Table 2). This stage was associated with the release of water and was confirmed by the endothermic peak at 35 °C recorded on the c-DTA curve (Figure 3a, Table 3) [36]. The second stage began at 127 °C with a maximum mass loss at 143 °C and contained a small weight loss of 9.45%. The third stage occurred at the temperature range of 166 °C to 269 °C (maximum mass change—241 °C). This stage was associated with the thermal decomposition of the component of the lozenge base. This fact was confirmed by the exothermic peak in the c-DTA curve (Figure 3b, Table 3). The third stage started at a temperature of 269 °C and was associated with a continued thermal degradation of the lozenge base. The maximum mass loss peak was 288 °C for the third stage. This stage also recorded an exothermic peak with a maximum of 306.8 °C on the c-DTA curve (Figure 3b, Table 3).

The thermogravimetry curve of the propolis lozenges showed that the decomposition onset is between pure propolis and lozenge base, with a value of 197.3 °C (Figure 4, Table 1). Figure 4 displays four stages of mass loss. The shape of the TG curve and the events maximum mass loss in DTG/D2TG are more similar to the curve of the lozenge base but also exhibit characteristics of the pure propolis curve. This indicates that the propolis is well-mixed within the lozenge formulation [37]. Moreover, the absence of a clear shift in the beginning of decomposition and the lack of additional peaks on the TG/DTG/D2TG/c-DTA curves recorded for propolis lozenge testify to the thermal compatibility of propolis with the ingredients used for the production of lozenges [9,38]. The first, second, third, and fourth stages of mass loss were 2.74%, 9.68%, 54.38%, and 31.21%, respectively (Figure 6a). The DTG and D2TG curves presented in Figure 4b exhibit four peaks corresponding to the thermogravimetry curve (Table 2). The first stage of mass loss occurred in the temperature range of 40 °C to 70 °C (maximum peak—59 °C). This stage is associated with water release and is observed in the lozenge base, accompanied by an endothermic peak with a maximum of 48 °C in the c-DTA curve (Figure 4a, Table 3) [36]. 

The second stage of mass loss occurred in the temperature range of 70 °C to 154 °C with a maximum peak at 132 °C. This peak is distinct from the corresponding peak in the lozenge base DTG curve. The reason for this difference is that this peak represents components from the lozenge base and pure propolis. The third stage of mass loss occurred in the temperature range of 154 °C to 260 °C, with the maximum peak at 227 °C. The last stage of mass loss began at 260 °C with a maximum mass loss peak at 285 °C. For the last two stages, exothermic peaks were recorded at 255 °C and 306 °C, respectively (Figure 4b, Table 2). These peaks are associated with the thermal decomposition of the sample.

#### 3.1.2. Influence of Stress Condition on the Tested Formulation

In the experiment, stress storage conditions were employed to evaluate propolis lozenges. For this purpose, physical factors such as higher temperature, relative humidity, and UV radiation were applied. The storage conditions of 40 °C/75%RH and 14 days were chosen, as they are recommended for preliminary testing of new drug formulations [6,16]. Additionally, the impact of UV radiation on the tested lozenges was examined [9]. Two exposure times, namely, 30 and 60 min, were utilized for this purpose. Thermal measurements (TG, DTG, D2TG, c-DTA), colorimetry analysis, and optical microscopic images were employed to assess the influence of the examined physical factors. 

Thermal analyses were conducted under the same conditions as the initial propolis lozenges. The obtained thermograms for the tested propolis lozenges treated under the prescribed physical storage conditions were similar to the thermogram of the initial propolis lozenges (Figure 4, Figure 5, Figure 6 and Figure 7). Minor differences were observed in samples stored under stress conditions (40 °C, 75% RH) for 14 days and subjected to 60 min of UV irradiation, specifically regarding the onset of decomposition. In these samples, the onset of decomposition shifted to lower temperatures by approximately 16 °C and 13 °C, respectively. Furthermore, in the DTG curves of propolis lozenges treated with physical factors, a reduction in weight loss during the third stage was observed, with an average decrease of 2%/min. compared to the initial propolis lozenges.

**Figure 8:**
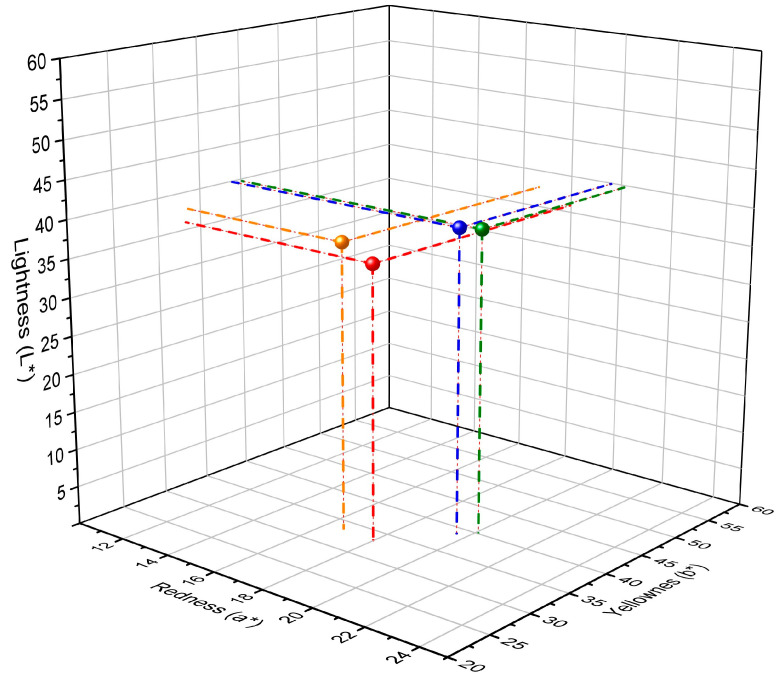
Analysis of colour in the 3D CIE L*a*b* space for initial propolis lozenges (green), propolis lozenges subjected to stress conditions (red), and UV irradiated 30 (blue) and 60 min (orange). Axes L*, a* and b* define the 3D colour space CIE.

Additionally, the most significant changes in the colorimetric analysis were observed for the same samples as in the thermal measurements. The location of colours in CIE L*a*b* space for the tested propolis lozenges was illustrated in Figure 8. The CIE L*a*b* parameters for propolis lozenges storage in stress conditions were different compared to the initial sample. For all samples treated, physical factors were observed as visible changes of colour. This fact indicates the ΔE parameter (Table 4). The changes in colour parameters were especially visible for propolis storage at a temperature of 40 °C, relative humidity of 75% for 14 days, and propolis lozenges UV radiated for 60 min (Figure 8, Table 4).

**Figure 9:**
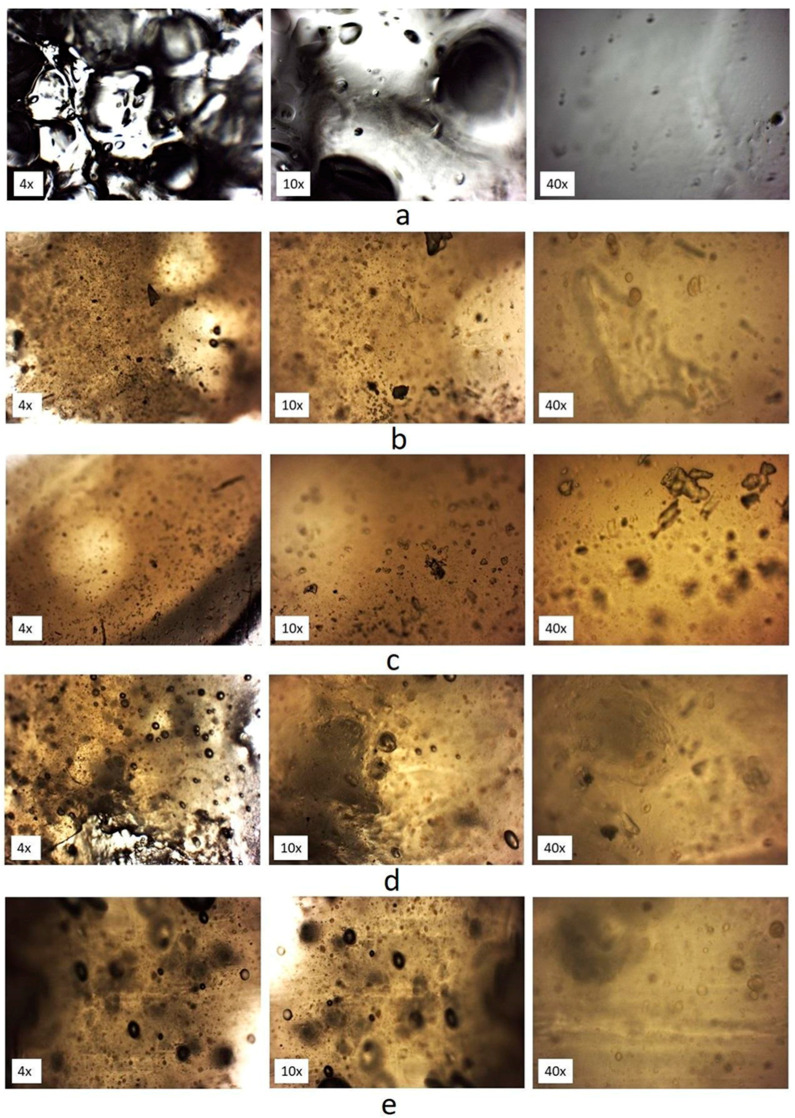
Optical microscopic images of (**a**) lozenge base; (**b**) initial propolis lozenge; (**c**) propolis lozenge stored in stress condition (40 °C/75%RH/14 days); (**d**) propolis lozenge UV-irradiated during 30 min and (**e**) 60 min.

For the base lozenges and tested propolis lozenges, optical microscope images were recorded (Figure 9). The most significant changes can be observed in relation to the photos of the lozenge base and lozenge containing propolis. After adding propolis to the formulation, we observe a clear change in the lozenge’s colour and the granularity’s presence. This is due to the good dispersion of propolis in the formulation. Analyzing the obtained microscopic images in quantitative terms, we observe slight differences in particle shape, size, and quantitative distribution. Most of the particles were round but also observed larger oval-shaped particles. They were particularly visible for propolis lozenge storage in stress conditions. The average number of particles present in the tested samples at 40× magnification was between 200 and 250 for particles of 40 to 50 µm. In turn, for particles with a size of 51–60 µm and 61–80 µm, these were the amounts of 60–80 and 40–50, respectively. However, it should be borne in mind that the optical microscopy method, despite being recommended by pharmacopeias, is one of the simplest methods of image analysis.

### 3.2. Microbiological Analysis of EEP and Prepared Lozenges

The sensitivity of four reference strains to EEP was assessed using the microdilution method. Table 5 shows the susceptibility of the tested strains based on the determined minimum inhibitory concentration (MIC) and minimum bactericidal concentration (MBC) values.

The used EEP at the concentrations of 50 µg/mL and 25 µg/mL showed bacteriostatic effects against the tested strains of oral streptococci. MBC values were in the range of 100–50 µg/mL of applied EEP. Lozenges with EEP were used for the further stage of this experiment. Lozenges with EEP were dissolved in 50 mL of artificial saliva. The concentration of EEP in the lozenge was 100 µg in 1 mL of artificial saliva. The results showed that the MIC and MBC of EEP-containing lozenges were lower than 100 µg/mL against the tested microbial strains, which correlates with previous results obtained for EEP. 

The most sensitive strains among those tested for EEP are *S. mitis* and *S. mutans*. On the other hand, *S. oralis* is less sensitive than *S. mitis* and *S. mutans*. Moreover, *S. salivarius* shows the lowest sensitivity to EEP among the tested strains.

All EEP concentrations tested showed statistically significant differences from the control group used with CHX 0.2% (*p* < 0.05), a gold standard antiplaque agent. To date, CHX has been recognized as the antimicrobial agent and the antiplaque agent at a concentration 0.2% [39,40,41,42]; that was the reason we used 0.2% solution of CHX with tested strains as a positive control. Photographs showing the plates together with the obtained results are presented in Table S1. No statistically significant differences were observed for the tested concentrations of EEP from 12.5 µg/mL compared to the control groups, i.e., (broth and bacteria) and (broth and bacteria and DMSO 0.1%), with *p* > 0.05 (Table S2). It demonstrated that a concentration of 50 µg/mL is necessary to inhibit growth, and 100 µg/mL is required for the bactericidal effect, with the lowest sensitivity observed in the *S. salivarius* strain.

The team led by Kashi T. [43] examined the ethanolic and water extracts of the Iranian propolis from the northeast area of Tehran against oral strains. They showed that MIC and MBC of ethanolic extract of the Iranian propolis were 250 μg/mL for *Streptococcus mutans* and 500 μg/mL for *Streptococcus salivarius*. In contrast, Barrientos L. [44] investigated the biological activity of Chilean propolis against the cariogenic bacteria *Streptococcus mutans* and *Streptococcus sobrinus*. They found that twenty samples of EEP from different regions of Chile inhibited the mutans streptococci growth and had MIC values ranging from 0.90 μg/mL to 8.22 μg/mL. Another study conducted by Koo H. [45] investigated the antibacterial activity of propolis from different regions (Northeastern, Southeastern, and Southern) of Brazil against *Streptococcus mutans*, *S. sobrinus* and *S. cricetus*. The ranges of MIC values were 50–400 μg/mL for *Streptococcus mutans* and 25–400 μg/mL for *Streptococcus sobrinus* and *Streptococcus cricetus*. As is well known, the chemical composition and pharmacological activity of propolis vary depending on the vegetation of the geographical area from which it originates. Polish propolis primarily comes from the leaf buds of poplar trees (*Populus nigra*), and the following flavonoids are commonly detected in EEP: chrysin, quercetin, kaempferol, pinocembrin, pinostrobin, pinobanksin, galangin, and apigenin, as well as phenolic and aromatic substances [46,47]. Propolis from different geographic regions in many countries shows antimicrobial activity but with different MIC and MBC values. We observed a large variation in MIC and MBC values compared to the same tested strain. For example, the antibacterial activity of the hydroalcoholic extract of S. mutans red propolis from Brazil expressed as MIC and MBC value is 292.97 µg/mL for MIC and 1171.87 µg/mL for MBC, respectively, while the MIC value for Anatolian propolis samples was evaluated by Uzel et al. as MIC = 8.0 µg/mL [48,49].

Our results indicated that the ethanolic extract of Polish propolis exhibits an antibacterial effect and demonstrated the MIC value for tested strains at the range of 25–50 µg/mL (Table 1). Comparing our findings with other in vitro studies, we noticed that propolis from different countries exhibits antibacterial effects against periodontal pathogenic bacteria, so Polish propolis demonstrates antibacterial efficacy on cariogenic bacteria [2,24,50,51]. 

Otręba et al. in a systematic review conducted in 2022 summarized the ranges of concentrations of MIC and MBC for different propolis extracts. It is worth noting that the ethanolic extract of propolis is more efficient than aqueous propolis extract during the examination of the antimicrobial activity [51]. 

Additionally, propolis shows antimicrobial activity against pathogens taking part in periodontal diseases. *Porphyromonas gingivalis* is a Gram-negative bacterium that has been implicated as a major etiological factor contributing to chronic periodontitis. Propolis is considered to be more effective against Gram-positive bacteria than Gram-negative because it can interact with lipids in the plasma membrane causing higher membrane permeability. Therefore, it reaches the cell membrane more easily in Gram-positive than in Gram-negative bacteria. According to Yoshimasu et al., propolis is effective against *Porphyromonas gingivalis*. His value of MIC is 64 µg/mL (broth) and 128 µg/mL (agar) for *Porphyromonas gingivalis* [2]. Clinical studies demonstrate that propolis causes regression of chronic periodontitis and gingivitis [52].

In a study conducted by Kolayli et al., all studied ethanolic extracts of propolis demonstrate good antibacterial activity against Gram-negative bacteria [53].

Overview, regarding our results and other in vitro and in vivo studies, propolis is a prospective natural product to treat and prevent periodontal diseases and caries, as well as dental plaque, caused by bacterial infections of the oral cavity.

However, some physical conditions could change the biological properties of propolis which have been demonstrated by Komosińska-Vassev K. et al. [54]. 

The authors showed that UV radiation decreased the scavenging activity of Polish propolis extract at concentrations of 7% and 10%. The stability of propolis is very important, taking into consideration the impact of the environmental factors on the quality of the prepared formulation. Thanks to this observation, we decided to study the impact of storage conditions on the proposed formulation to aim to provide the highest antibacterial activity of lozenges. For each tested standard strain, statistically significant differences were observed in the MIC and MBC values obtained for the tested propolis lozenges subjected to stress conditions (Tables S3–S7). In each case, higher absorbance values were obtained for the unstressed propolis lozenges compared to the lozenges treated with the stress factor.

Temperature and humidity fluctuations in the 14-day test as well as UV radiation destabilized EEP and affected the final activity against microbial (Table S8). Comparing the absorbance values obtained, it can be concluded that the stress factor, such as 30 min of UV radiation, had the most potent effect on the reduction of antimicrobial activity. The most sensitive strain to EEP was *S. mitis*, which confirms the results of previous tests and observations. For most tested strains, there is a statistically significant difference in observed absorbance value between the effect of UV radiation and the 14-day incubation period under increased humidity and temperature conditions. Interestingly, in the case of some strains, there are no statistically significant differences (*p* > 0.05) (Tables S3–S7) between temperature and humidity fluctuations and exposure to UV radiation, although both factors affect the final activity of EEP, reducing it. Thus, lozenges exposed to stress conditions showed less activity against *Staphylococcus* in the oral cavity. As our results showed lozenges with EEP exerted the bacteriostatic and bactericidal effect (MIC and MBC < 100 µg/mL) compared to all the tested strains (Table 5).

## 4. Conclusions

This conducted study demonstrated the antibacterial effects of the ethanolic extract of Polish propolis concerning cariogenic bacteria. The evaluation of the antibacterial effect of propolis and prepared lozenges indicates a potential role of ethanolic extract of Polish propolis in prophylaxis and therapeutic strategy decreasing the accumulation of dental plaque. Therefore, it is worth highlighting that propolis may play an important role in the management of dental health and brings advantages in the maintenance of the oral cavity in healthy conditions. The proposed formulation of ethanolic extract of propolis as lozenges composed of gelatin–glycerol mass with the addition of xylitol might be useful. Chewable lozenges are a good alternative to using the carrier of propolis. Appropriate and acceptable dosage form has a huge impact on effective therapy. The proposed dosage form is intended to be slowly dissolved in the mouth allowing for a longer effect of propolis on oral pathogens. In the conducted experiment, the stress storage conditions of 40 °C and 14 days were used to evaluate the propolis lozenge, because these conditions are recommended for testing new formulations. Our findings showed that lozenges are a stable option and ethanolic extract of propolis retains its antibacterial activity. Therefore, the obtained results indicate that chewable lozenge might be a promising dosage form with a cariostatic agent such as propolis.

## Figures and Tables

**Table 1:** Parameters of TG curves recorded for the tested samples.

Sample	Onset [°C]	Mid [°C]	Inflection [°C]	End [°C]	Mass Change [%]
Pure propolis	211.4	312.4	316.2	403.6	−41.01
Lozenge base	189.1	263	289.9	319.9	−69.02
Propolis lozenge	197.3	249.1	228.6	286.2	−71.25
Propolis lozenge exposed to 40 °C/75%HR/14 days	181.4	247.6	225.3	302.7	−64.06
Propolis lozenge exposed to UV/30 min	188	248	232.6	304.2	−75.57
Propolis lozenge exposed to UV/60 min	184.7	251.7	236.1	308.4	−63.38

**Table 2:** Characteristic parameters of DTG, and D2TG curves of the tested propolis.

Sample	Stage	DTG		D2TG	
		Peak [°C]	Mass Change [%/min.]	Peak Min. [°C]	Peak Max. [°C]
Pure propolis	I	145.5	−1.1	125.8	145.9
	II	206.4	−2.52	183.4	219.4
	III	348.5	−3.4	256.3	364.8
	IV	-	-	-	-
Lozenge base	I	56.9	−0.81	42.7	64.2
	II	143.8	−1.66	137.8	157.5
	III	241.4	−7.18	199.3	252.5
	IV	288.3	−7.35	278.3	300.1
Propolis lozenge	I	59.3	−0.98	49	67.4
	II	132.8	−1.68	94.4	142
	III	227.2	−9.24	208.9	243.4
	IV	285.7	−6.89	274.6	297
Propolis lozenge exposed to 40 °C/75%HR/14 days	I	55.1	−1.18	45.3	62.8
	II	122.2	−1.91	112.1	131
	III	227.2	−7.09	195.9	241.9
	IV	284.3	−6.26	271.4	295.1
Propolis lozenge exposed to UV/30 min	I	59.1	−0.63	43.3	63.5
	II	104.9	−0.88	91.5	113.8
	III	233.2	−8.44	206.6	249.7
	IV	286.2	−7.19	275.6	298.5
Propolis lozenge exposed to UV/60 min	I	59.8	−1.11	39	73
	II	109.4	−1.74	73	135
	III	234.7	−6.96	135	261
	IV	286	−6.08	261	375

**Table 3:** Characteristic parameters of c-DTA curves of the tested propolis. Endo-endothermic reaction, Exo-exothermic reaction.

Sample	Stage	Onset [°C]	Peak [°C]	Area [K × s]	Type of Reaction
Pure propolis	I	41	47.1	138.14	endo
	II	189	226.6	19.1399	exo
	III	333.6	355.8	57.7894	exo
Lozenge base	I	32	35.2	128.921	endo
	II	245.2	262.6	26.7208	exo
	III	296.4	306.8	33.9138	exo
Propolis lozenge	I	43	48.7	214.585	endo
	II	228.7	255.6	95.5586	exo
	III	285	306	52.9155	exo
Propolis lozenge exposed to 40 °C/75%HR/14 days	I	43	57.2	123.59	endo
	II	220	255.3	68.1004	exo
	III	283.8	303.8	49.8703	exo
Propolis lozenge exposed to UV/30 min	I	30	33.4	474.942	endo
	II	242.1	263.9	61.1707	exo
	III	294.2	308.6	47.8503	exo
Propolis lozenge exposed to UV/60 min	I	35	40.4	226.767	endo
	II	249.4	259.8	31.603	exo
	III	297.4	309.3	24.4174	exo

**Table 4:** Analysis of colour in the CIE L*a*b* space, total colour difference (ΔE), and browning index (BI) parameters for propolis lozenges stored in different conditions.

Propolis Lozenges	L* [±SD]	a* [±SD]	b* [±SD]	ΔE [±SD]	BI [±SD]
Initial	39.72 [±0.09]	20.10 [±0.05]	40.10 [±0.13]	-	241.52 [±0.13]
40 °C/75% RH/14 days	35.97 [±0.31]	17.98 [±0.31]	33.24 [±0.01]	8.10 [±0.31]	208.87 [±0.31]
UV/30 min	39.93 [±0.06]	19.60 [±0.02]	38.88 [±0.08]	1.34 [±0.08]	226.57 [±0.08]
UV/60 min	37.69 [±0.48]	16.65 [±0.14]	33.54 [±0.30]	7.69 [±0.30]	193.82 [±0.30]

The results are presented as mean ± SD [n = 8]; The results were considered statistically significant when *p* < 0.05.

**Table 5:** MIC and MBC values of EEP on the tested microorganism, prepared lozenges, and controls.

Tested Bacteria	EEP		Lozenges		Lozenges after a Conditioning Test		Control Positive		Control Negative	
	MIC [µg/mL]	MBC [µg/mL]	MIC [µg/mL]	MBC [µg/mL]	MIC [µg/mL]	MBC [µg/mL]	MIC [µg/mL]	MBC [µg/mL]	MIC*	MBC*
*Streptococcus* *mutans*	25	50	<100	<100	<100	<100	<2000	<2000	1 × 10^5^ CFU/mL	1 × 10^5^ CFU/mL
*Streptococcus* *salivarius*	50	100	<100	<100	<100	<100	<2000	<2000	1 × 10^5^ CFU/mL	1 × 10^5^ CFU/mL
*Streptococcus* *mitis*	25	50	<100	<100	<100	<100	<2000	<2000	1 × 10^5^ CFU/mL	1 × 10^5^ CFU/mL
*Streptococcus* *oralis*	50	50	<100	<100	<100	<100	<2000	<2000	1 × 10^5^ CFU/mL	1 × 10^5^ CFU/mL

MIC* and MBC* have been expressed as a colony-forming unit (CFU) in one milliliter determined by a densitometer.

## Data Availability

Not applicable.

## Supplementary Materials

The following supporting information can be downloaded at: https://www.mdpi.com/article/10.3390/pharmaceutics15061768/s1, Table S1: Photos presenting 96-well plates and plates with broth made to determine the minimum bactericidal concentration for EEP; Table S2: Results of main effect of ANOVA analysis for MIC of tested EEP concentrations; Table S3: Results of main effect of ANOVA analysis for MIC values of tested lozenges; Table S4: Results of Fisher’s LSD Test for *S. mitis* MIC values of tested lozenges; Table S5: Results of Fisher’s LSD Test for *S. mutans* MIC values of tested lozenges; Table S6: Results of Fisher’s LSD Test for *S. oralis* MIC values of tested lozenges; Table S7: Results of Fisher’s LSD Test for *S. salivarius* MIC values of tested lozenges; Table S8: Photos presenting 96-well plates and plates with broth made to determine the minimum bactericidal concentration for prepared lozenges with EEP.
