# Supplementary material for: Propolis as a Cariostatic Agent in Lozenges and Impact of Storage Conditions on the Stability of Propolis
Source: Pharmaceutics. 2023 Jun 19;15(6):1768. doi: 10.3390/pharmaceutics15061768 (PMC10303535; doi:10.3390/pharmaceutics15061768)
Supplement: Supplementary file 1 [file pharmaceutics-15-01768-s001.zip › pharmaceutics-2390681-supplementary.pdf]

pharmaceutics

Supplementary Materials

Propolis as a Cariostatic Agent in Lozenges and Impact of Storage Conditions on the Stability of Propolis

Anna Kurek-Górecka [1,*], Paweł Ramos [2], Małgorzata Kłósek [3], Elżbieta Bobela [3], Zenon P. Czuba [3], Radosław Balwierz [4] and Paweł Olczyk [1]

1 Department of Community Pharmacy, Faculty of Pharmaceutical Sciences in Sosnowiec, Medical University of Silesia in Katowice, Kasztanowa 3, 41-200 Sosnowiec, Poland; polczyk@sum.edu.pl
2 Department of Biophysics, Faculty of Pharmaceutical Sciences in Sosnowiec, Medical University of Silesia in Katowice, Jedności 8, 41-200 Sosnowiec, Poland; pawelramos@sum.edu.pl
3 Department of Microbiology and Immunology, Faculty of Medical Sciences in Zabrze, Medical University of Silesia in Katowice, Jordana 19, 41-808 Zabrze, Poland; mklosek@sum.edu.pl (M.K.); ebobela@sum.edu.pl (E.B.); zczuba@sum.edu.pl (Z.P.C.)
4 Institute of Chemistry, University of Opole, Oleska 48, 45-052 Opole, Poland; radoslaw.balwierz@uni.opole.pl
* Correspondence: akurekgorecka@sum.edu.pl

Table of content	p.
**Table S1.** Photos presenting 96-well plates and plates with broth made to determine the minimum bactericidal concentration for EEP.	2
**Table S2.** Results of main effect of ANOVA analysis for MIC of tested EEP concentrations.	4
**Table S3.** Results of main effect of ANOVA analysis for MIC values of tested lozenges.	4
**Table S4.** Results of Fisher's LSD Test for *S. mitis* MIC values of tested lozenges.	5
**Table S5.** Results of Fisher's LSD Test for *S. mutans* MIC values of tested lozenges.	6
**Table S6.** Results of Fisher's LSD Test for *S. oralis* MIC values of tested lozenges.	7
**Table S7.** Results of Fisher's LSD Test for *S. salivarius* MIC values of tested lozenges.	8
**Table S8.** Photos presenting 96-well plates and plates with broth made to determine the minimum bactericidal concentration for prepared lozenges with EEP.	9

Table S1. Photos presenting 96-well plates and plates with broth made to determine the minimum bactericidal concentration for EEP.

Bacteria	Photos of the plate presenting strains bacteria tested	Photos of the 96-well microtiter plates used to determination of the Minimum Inhibitory Concentration and Minimum Bactericidal Concentration of EEP	
Streptococcus mitis	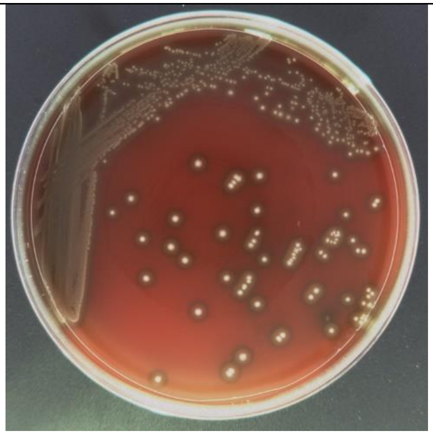	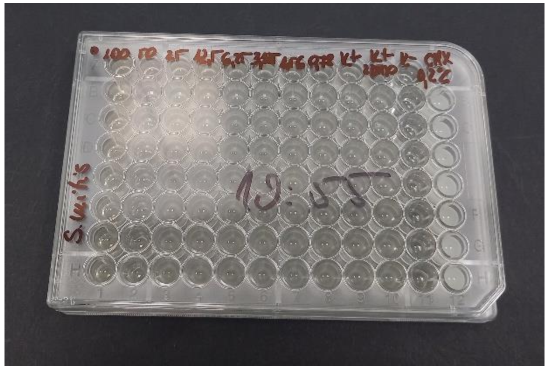	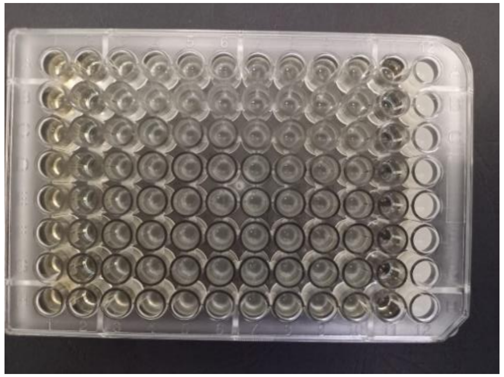
Streptococcus mutans	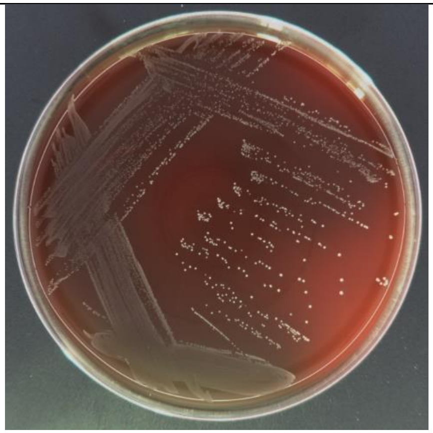	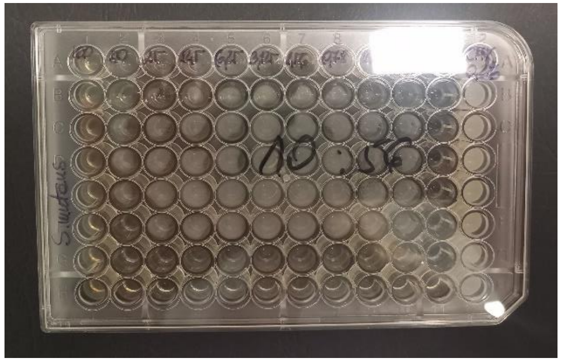	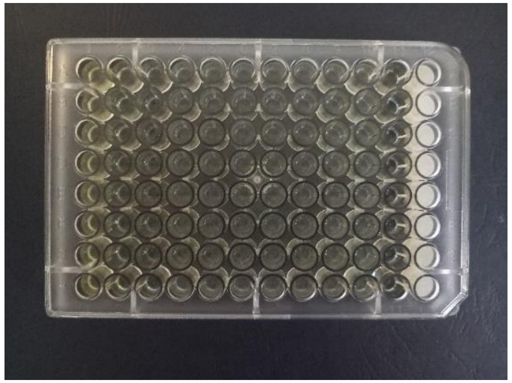

Streptococcus oralis	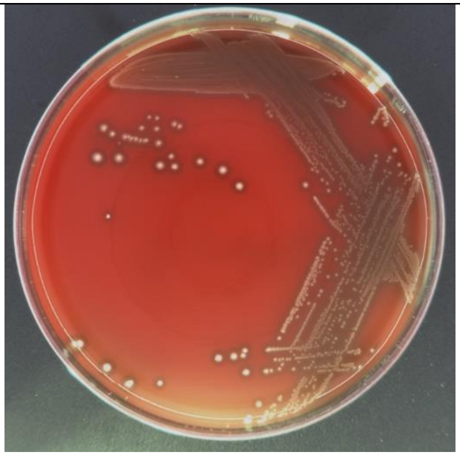	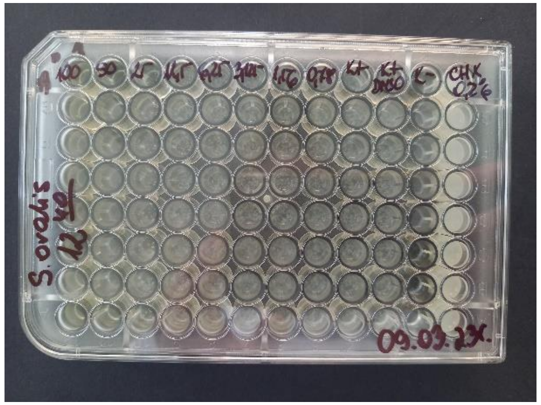	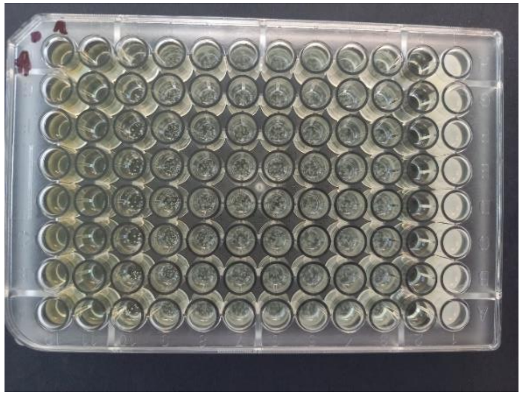
Streptococcus salivarius	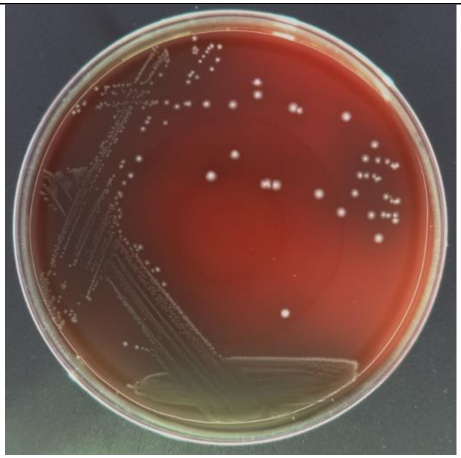	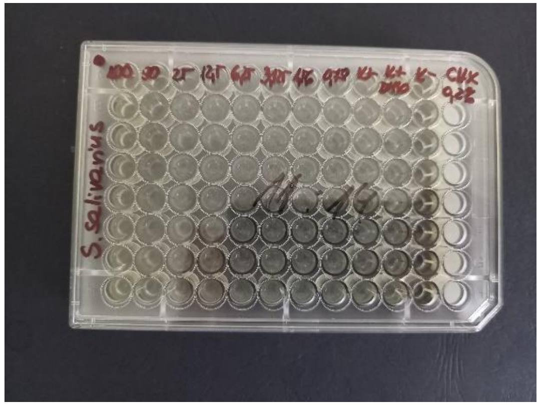	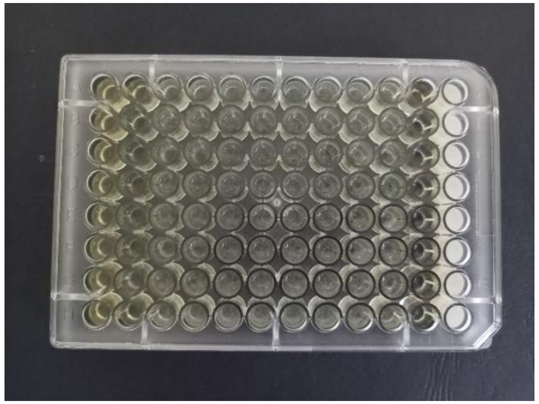

Table S2. Results of main effect of ANOVA analysis for MIC of tested EEP concentrations.

Effect	Multivariate Tests of Significance<br>Sigma-restricted parameterization<br>Effective hypothesis decomposition					
	**Test**	**Value**	**F**	**Effect df**	**Error df**	**p**
Intercept	Wilks	0,001428	14160,06	4	81,0000	0,00
Formulation	Wilks	0,000079	76,57	44	311,8403	0,00

Results marked in red are statistically significant at the level $p \leq 0.05$.

Table S3. Results of main effect of ANOVA analysis for MIC values of tested lozenges.

Effect	Multivariate Tests of Significance0<br>Sigma-restricted parameterization<br>Effective hypothesis decomposition					
	**Test**	**Value**	**F**	**Effect df**	**Error df**	**p**
Intercept	Wilks	0,004078	4762,232	4	78,0000	0,00
Formulation	Wilks	0,003115	48,173	24	273,3195	0,00

* Results marked in red are statistically significant at the level $p \leq 0.05$.

Table S4. Results of Fisher's LSD Test for *S. mitis* MIC values of tested lozenges.

	LSD test; variable S. mitis<br>Probabilities for Post Hoc Tests Error: Between MS = .00048. df = 49.000							
**Cell No.**	**Formulation**	**{1}<br>.29812**	**{2}<br>.28412**	**{3}<br>.27088**	**{4}<br>.35012**	**{5}<br>.04212**	**{6}<br>.06425**	**{7}<br>.04037**
1	PT		0.206348	0.016107	0.000018	0.000000	0.000000	0.000000
2	PT/HT	0.206348		0.231311	0.000000	0.000000	0.000000	0.000000
3	PT/UV30m	0.016107	0.231311		0.000000	0.000000	0.000000	0.000000
4	PT/UV60m	0.000018	0.000000	0.000000		0.000000	0.000000	0.000000
5	Control (saliva & bacteria)	0.000000	0.000000	0.000000	0.000000		0.048454	0.873476
6	Control (tablets WP & bacteria)	0.000000	0.000000	0.000000	0.000000	0.048454		0.033782
7	Control (saliva)	0.000000	0.000000	0.000000	0.000000	0.873476	0.033782	

Results marked in red are statistically significant at the level $p \leq 0.05$.

Table S5. Results of Fisher's LSD Test for *S. mutans* MIC values of tested lozenges.

	LSD test; variable S. mutans<br>Probabilities for Post Hoc Tests Error: Between MS = .00267. df = 49.000							
Cell No.	Formulation	{1}<br>1.3363	{2}<br>1.1121	{3}<br>1.0305	{4}<br>1.1420	{5}<br>.05487	{6}<br>.08238	{7}<br>.05237
1	PT		0.000000	0.000000	0.000000	0.000000	0.000000	0.000000
2	PT/HT	0.000000		0.002725	0.253465	0.000000	0.000000	0.000000
3	PT/UV30m	0.000000	0.002725		0.000078	0.000000	0.000000	0.000000
4	PT/UV60m	0.000000	0.253465	0.000078		0.000000	0.000000	0.000000
5	Control (saliva & bacteria)	0.000000	0.000000	0.000000	0.000000		0.292681	0.923359
6	Control (tablets WP & bacteria)	0.000000	0.000000	0.000000	0.000000	0.292681		0.251511
7	Control (saliva)	0.000000	0.000000	0.000000	0.000000	0.923359	0.251511	

Results marked in red are statistically significant at the level $p \leq 0.05$.

Table S6. Results of Fisher's LSD Test for *S. oralis* MIC values of tested lozenges.

Cell No.	Formulation	LSD test; variable S. oralis Probabilities for Post Hoc Tests Error: Between MS = .00116. df = 49.000						
		{1} 1.2576	{2} 1.0605	{3} .98200	{4} 1.1495	{5} .05475	{6} .11000	{7} .05275
1	PT		0.000000	0.000000	0.000000	0.000000	0.000000	0.000000
2	PT/HT	0.000000		0.000029	0.000004	0.000000	0.000000	0.000000
3	PT/UV30m	0.000000	0.000029		0.000000	0.000000	0.000000	0.000000
4	PT/UV60m	0.000000	0.000004	0.000000		0.000000	0.000000	0.000000
5	Control (saliva & bacteria)	0.000000	0.000000	0.000000	0.000000		0.002121	0.906979
6	Control (tablets WP & bacteria)	0.000000	0.000000	0.000000	0.000000	0.002121		0.001507
7	Control (saliva)	0.000000	0.000000	0.000000	0.000000	0.906979	0.001507	

Results marked in red are statistically significant at the level $p \leq 0.05$.

Table S7. Results of Fisher's LSD Test for *S. salivarius* MIC values of tested lozenges.

	LSD test; variable S. salivarius<br>Probabilities for Post Hoc Tests Error: Between MS = .00583. df = 49.000							
**Cell No.**	**Formulation**	**{1}<br>1.3731**	**{2}<br>1.1195**	**{3}<br>1.0253**	**{4}<br>1.1885**	**{5}<br>.05262**	**{6}<br>.09975**	**{7}<br>.05237**
1	PT		0.000000	0.000000	0.000014	0.000000	0.000000	0.000000
2	PT/HT	0.000000		0.017122	0.076924	0.000000	0.000000	0.000000
3	PT/UV30m	0.000000	0.017122		0.000088	0.000000	0.000000	0.000000
4	PT/UV60m	0.000014	0.076924	0.000088		0.000000	0.000000	0.000000
5	Control (saliva & bacteria)	0.000000	0.000000	0.000000	0.000000		0.223073	0.994803
6	Control (tablets WP & bacteria)	0.000000	0.000000	0.000000	0.000000	0.223073		0.220665
7	Control (saliva)	0.000000	0.000000	0.000000	0.000000	0.994803	0.220665	

Results marked in red are statistically significant at the level $p \leq 0.05$.

Table S8. Photos presenting 96-well plates and plates with broth made to determine the minimum bactericidal concentration for prepared lozenges with EEP.

**Bacteria**	**Photos of the 96-well microtiter plates used to determination of the Minimum Inhibitory Concentration and Minimum Bactericidal Concentration of EEP**	
Streptococcus mitis	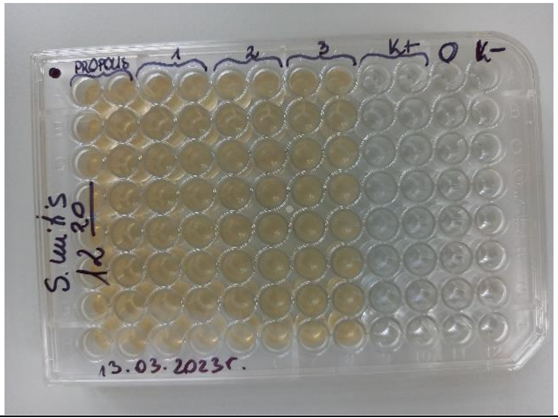	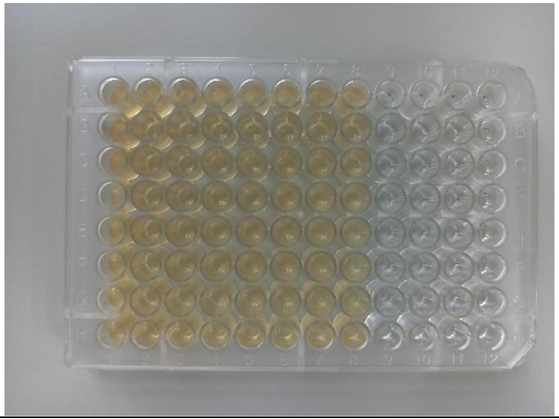
Streptococcus mutans	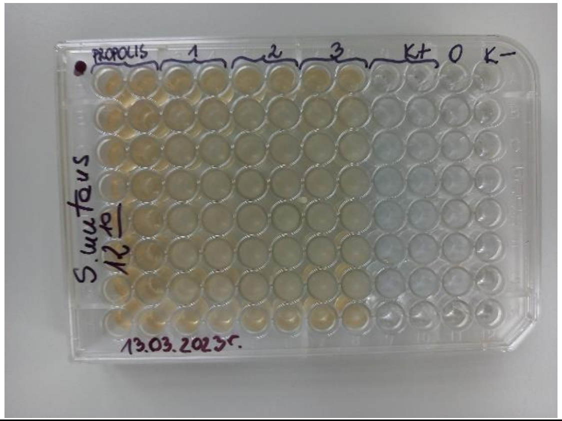	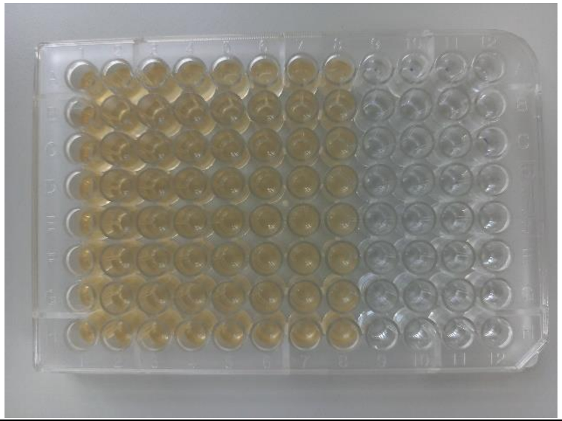

Streptococcus oralis	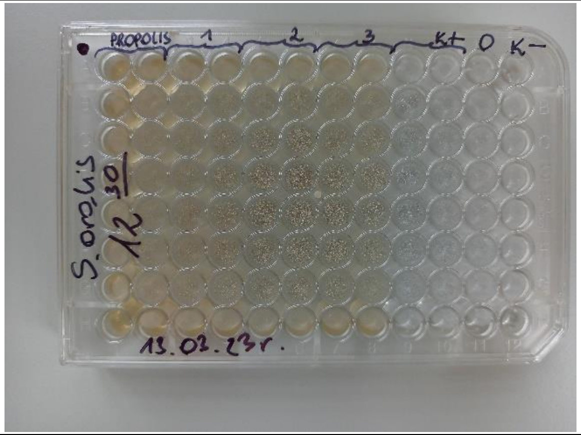	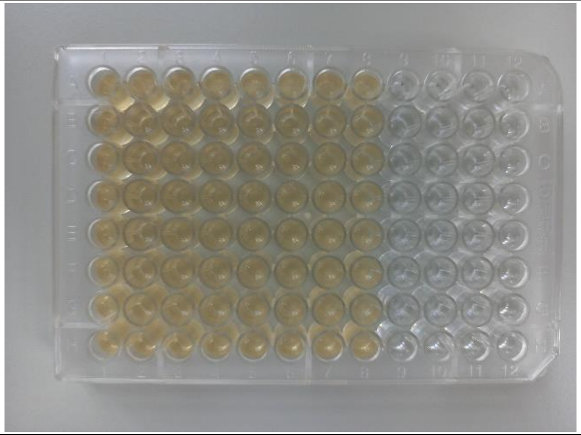
Streptococcus salivarius	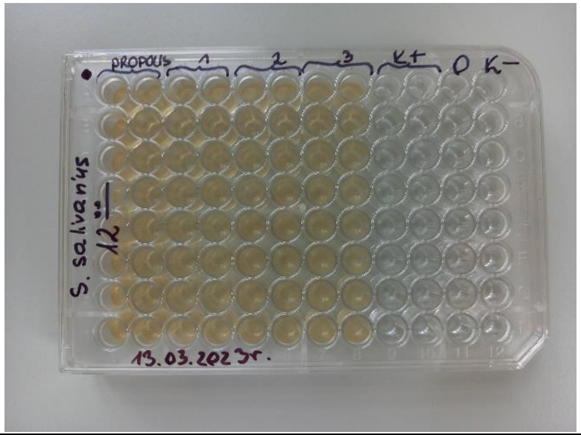	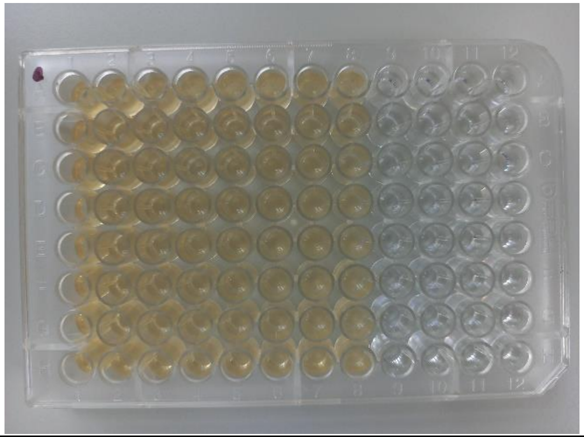

Disclaimer/Publisher's Note: The statements, opinions and data contained in all publications are solely those of the individual author(s) and contributor(s) and not of MDPI and/or the editor(s). MDPI and/or the editor(s) disclaim responsibility for any injury to people or property resulting from any ideas, methods, instructions or products referred to in the content.
